# Leukocytoclastic Vasculitis Associated with a New Anticoagulant: Rivaroxaban

**DOI:** 10.4274/tjh.2016.0353

**Published:** 2017-03-01

**Authors:** Nuri Barış Hasbal, Taner Baştürk, Yener Koç, Tuncay Sahutoğlu, Feyza Bayrakdar Çağlayan, Abdülkadir Ünsal

**Affiliations:** 1 Şişli Hamidiye Etfal Training and Research Hospital, Clinic of Nephrology, İstanbul, Turkey

**Keywords:** Vasculitis, Anticoagulants, Rivaroxaban

## TO THE EDITOR,

Rivaroxaban, an oral direct factor Xa inhibitor, is one of the non-vitamin K antagonist anticoagulants and has been approved for various thrombotic diseases. Here we present a patient who developed leukocytoclastic vasculitis (LCV) associated with rivaroxaban as a rare non-bleeding side effect.

**Figure 1:**
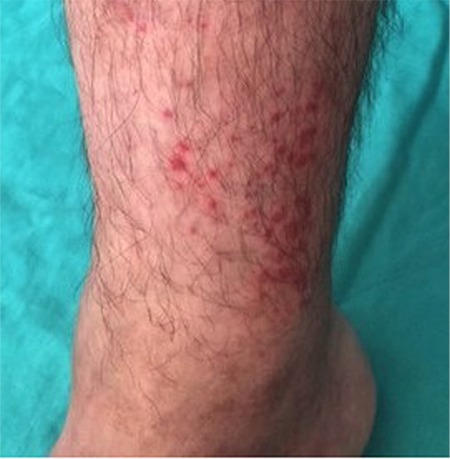
Non-blanching palpable purpura is seen on the right lower leg.

A 28-year-old man who was being treated with diltiazem (60 mg/day) and oral methylprednisolone (32 mg on alternate days) (6^th^ month of Pozzi protocol [1]) for IgA nephropathy was admitted to our hospital with bilateral lower extremity non-blanching palpable purpura that occurred 10 days following the addition of 20 mg of rivaroxaban once daily for acute deep venous thrombosis in the right popliteal vein by another physician. There was no significant finding in the physical examination except for purpura. The complete blood count, metabolic panel, urine analysis, coagulation studies, infectious serologies, rheumatologic work-up, and serum immunoglobulin E level were all within normal limits. Rivaroxaban was replaced with subcutaneous enoxaparin sodium at 6000 IU twice a day, and the skin lesions disappeared within 1 week. Two weeks later, the patient was prescribed rivaroxaban at 10 mg a day again by the same physician who was following the patient for deep venous thrombosis because of the rarity of LCV due to rivaroxaban in the literature. Bilateral lower extremity purpura (Figure 1) reoccurred within 3 days of retreatment and a skin biopsy revealed neutrophil-predominant infiltrations within and surrounding the dermal small vessels, nuclear dust, vessel wall damage, erythrocyte extravasation, and fibrin deposition concurrent with vasculitis. Rivaroxaban was discontinued and enoxaparin was administered again, and the skin lesions resolved. The patient was in a clinically steady state for IgA nephropathy during the two episodes of vasculitis.

LCV is associated with the deposition of the immune complex in small vessels that brings about loss of vessel wall integrity and extravasation of erythrocytes by immune response resulting in purpura. Although drugs and infections are the most common etiologies for LCV, idiopathic forms of the disease account for approximately half of all cases [2]. Connective tissue diseases, other systemic diseases, and hematologic or solid organ malignancies are other remaining causes of LCV [3]. The interval between administration of the suspected agent and the onset of symptoms is variable, symptoms mostly occur 7 to 10 days after exposure. Treatment of LCV starts with cessation of the causative drug and palliation of symptoms after systemic involvement is excluded. Systemic therapies such as colchicine, dapsone, corticosteroids, and some other immunosuppressive medications are used for managing serious and refractory disease [3,4].

There is only one similar report in the literature, from Chaaya et al., in which they presented a 68-year-old male patient with multiple comorbidities who developed signs of LCV after 7 days of rivaroxaban treatment due to deep venous thrombosis [5]. In that report, the findings of LCV disappeared within 1 week following the discontinuation of rivaroxaban and allopurinol plus a short course of intravenous methylprednisolone.

In conclusion, this report is the second case of rivaroxaban-associated LCV in the literature and this adverse event should be included in the list of significant adverse reactions to rivaroxaban.

All procedures performed in this study involving human participants were in accordance with the ethical standards of the institutional research committee and the 1964 Helsinki Declaration and its later amendments or comparable ethical standards.
